# Letter to the Editor: A commentary on ‘Prognostic significance of preoperative nutritional status for postoperative acute kidney injury in older patients undergoing major abdominal surgery: a retrospective cohort study’

**DOI:** 10.1097/JS9.0000000000001697

**Published:** 2024-05-22

**Authors:** Yiqi Huang, Yiming Jin, Feng Jin, Sanmeizi Xie

**Affiliations:** a Department of Nephrology, Shaoxing Second Hospital; b Intensive Care Unit, Shaoxing Second Hospital, Shaoxing, Zhejiang, People’s Republic of China

*Dear Editor,*

Acute kidney injury (AKI) is a common complication in elderly patients after abdominal surgery, with an incidence ranging from 3 to 35%, leading to serious complications^1^. Identifying risk factors for AKI in elderly patients is essential for prevention and treatment. The preoperative nutritional status is a crucial parameter that reflects the patient’s postoperative prognosis, as evidenced by numerous surgical studies^2,3^, but there is limited research on this for major abdominal surgeries. The retrospective cohort study conducted by Sun *et al*. examined the prognostic significance of preoperative nutritional status for postoperative AKI in older patients undergoing major abdominal surgery. The findings indicated that poor nutritional status has an independent association with an elevated risk of AKI in older patients following major abdominal surgery, and including nutritional indices to predict AKI models improved accuracy^4^.

However, the limitations of this study also need to be considered. Of note, urine output serves as a non-invasive and pivotal indicator of alterations in kidney function, but the aforementioned study failed to incorporate the perioperative period urine output, particularly postoperative urine output, as a variable. After undergoing surgery, it is advisable to monitor urine output. Our observations indicate that certain individuals may exhibit a slight elevation in serum creatinine (SCR) levels within 1 week following surgery, yet not fulfilling the criteria for AKI. Should there be a continued decrease in 24-h urine output, AKI may manifest up to 1 week after surgery, potentially resulting in the underdiagnosis of AKI patients.

Secondly, the exclusion criteria established by Sun *et al*. failed to evaluate the presence of AKI in patients prior to surgery. Given that elderly individuals are at a higher risk for AKI due to age-related physiological changes^5^, it is recommended to monitor SCR levels on two separate occasions before surgery to rule out preoperative AKI.

Thirdly, Sun and his team used the most recent SCR level measured within 7 days prior to surgery as the baseline creatinine value. We hypothesize that elderly patients undergoing major abdominal surgery generally experience a decline in food and exercise intake, resulting in a reduction of creatinine generation. Consequently, the SCR level during this period may reach its nadir. The study conducted by Siew *et al*.^6^ demonstrated that utilizing the lowest SCR as the baseline creatinine value may lead to an overestimation of AKI incidence. Therefore, the baseline creatinine level should be defined as the average of all SCR values within 7 days prior to surgery. This approach effectively mitigates bias associated with using a single value as the baseline SCR level.

Finally, elderly patients undergoing major abdominal surgery may experience challenges in tolerating acute and short-term pain, necessitating the administration of painkillers or pain pumps, which may increase the risk of AKI^7^. To mitigate potential confounding factors, we propose excluding patients who developed AKI as a result of painkiller usage from the study sample.

Additionally, we propose a novel idea: the potential replacement of albumin with prealbumin in the aforementioned three nutritional indices. The half-life of albumin spans 18–20 days, whereas that of prealbumin is merely 1.9 days^8^. Considering the diminished dietary intake among elderly patients prior to surgery, prealbumin proves to be a more accurate indicator for evaluating preoperative nutritional status in comparison to albumin. Nevertheless, the replacement of albumin with prealbumin in nutritional indices requires validation through a comprehensive prospective study.

Overall, this retrospective study presents convincing evidence that preoperative malnutrition serves as an independent risk factor for AKI in elderly patients undergoing major abdominal surgery. The development of innovative AKI prediction models may aid healthcare providers in establishing early warning systems and timely screening protocols for high-risk populations of AKI following major abdominal surgery, ultimately improving the prognosis of elderly patients. Consequently, we advocate for the inclusion of preoperative nutritional assessment as an essential component of risk stratification for AKI in elderly patients undergoing major abdominal surgery.

## Ethical approval

Not applicable.

## Consent

Not applicable.

## Sources of funding

Not applicable.

## Author contribution

Y.H., Y.J., F.J., and S.X.: conceptualization, methodology, validation, formal analysis, investigation, resources, data curation, writing – original draft, writing – review and editing, supervision, and project administration.

## Conflicts of interest disclosure

The authors declare that there are no conflicts of interest regarding the publication of this paper.

## Research registration unique identifying number (UIN)

Not applicable.

## Guarantor

Yiqi Huang, Yiming Jin, Feng Jin, and Sanmeizi Xie.

## Data availability statement

Data sharing is not applicable to this article as no new data were created or analyzed in this study.

## Provenance and peer review

Not applicable.
